# Detecting Residual Root Canal Filling Material After Retreatment: Cone-Beam Computed Tomography and Digital Microscopy Compared with Microcomputed Tomography

**DOI:** 10.3390/dj14060318

**Published:** 2026-05-22

**Authors:** Mohamad Alouda, Samar Akil, Mohammad Tamer Abbara, Ammar Eid, Imad-Addin Almasri, Yasser Alsayed Tolibah, Ziad D. Baghdadi

**Affiliations:** 1 Department of Endodontics, Damascus University, Damascus P.O. Box 3062, Syria; mhd98.ouda@damascusuniversity.edu.sy (M.A.); d_samar_al@hotmail.com (S.A.); 2 Department of Endodontics, University of Kalamoon, Damascus P.O. Box 3062, Syria; tamerabbara@gmail.com; 3 Department of Endodontics, International University for Science & Technology, Damascus P.O. Box 3062, Syria; ammarendo89@gmail.com; 4 Applied Statistics Department, Damascus University, Damascus P.O. Box 3062, Syria; imad_almasri27@yahoo.com; 5 Department of Pediatric Dentistry, Faculty of Dentistry, Damascus University, Damascus P.O. Box 3062, Syria; yasseralsayedtolibah@gmail.com; 6 Department of Preventive Dental Sciences, Division of Pediatric Dentistry, Dr. Gerald Niznick College of Dentistry, University of Manitoba, Winnipeg, MB R3E 0W2, Canada; 7 Centre for Community Oral Health, Dr. Gerald Niznick College of Dentistry, University of Manitoba, P131B, 780 Bannatyne Avenue, Winnipeg, MB R3E 0W2, Canada; 8 TopSmiles Pediatric Dentistry & Orthodontics, 246 St. Anne’s Road, Winnipeg, MB R2M 3A4, Canada; 9 Butterfly Dental Group, Winnipeg, MB R3Y 1P5, Canada; 10 Children’s Dental Centre, 1630 Ness Ave, Winnipeg, MB R3J 3X1, Canada

**Keywords:** root canal retreatment, cone-beam computed tomography, micro-CT, digital microscopy, calcium silicate-based sealer

## Abstract

**Background/Objectives**: Reliable detection of residual root canal filling material after retreatment is essential for comparing retreatment protocols. However, available methods quantify different clinical–physical dimensions and may not yield comparable estimates. This in vitro study compared cone-beam computed tomography (CBCT) and digital microscopy (DGM) for detecting residual obturation material after retreatment, using microcomputed tomography (micro-CT) as the reference standard. **Methods**: Fifteen extracted human mandibular premolars with single, straight canals were instrumented, obturated with gutta-percha and a calcium silicate-based sealer (AH Plus Bioceramic), and retreated with ProTaper Universal Retreatment files. Residual material was assessed in the coronal, middle, and apical thirds using CBCT (voxel size 0.10 mm), micro-CT (voxel size 60 µm), and DGM after longitudinal root splitting. Surface-based (DGM) and volumetric (CBCT and micro-CT) outcomes were analyzed separately using Wilcoxon signed-rank tests, diagnostic accuracy metrics (sensitivity, specificity, predictive values), and Cohen’s kappa for agreement. **Results**: DGM showed low median residual surface percentages across thirds (0.34–1.52%), whereas CBCT yielded higher median residual volume percentages (10.20–14.20%) than micro-CT (3.27–5.04%). The difference in the middle third between CBCT and micro-CT remained significant after Bonferroni correction (*p* = 0.002). For binary detection, CBCT showed higher sensitivity but lower specificity (overclassification of positive thirds), whereas DGM showed high specificity but limited sensitivity in the coronal and middle thirds. **Conclusions:** Within the limitations of this laboratory study, micro-CT was the most reliable reference method. CBCT tended to overestimate residual material, suggesting that clinical decisions based solely on CBCT may lead to unnecessary retreatment. DGM underestimated remnants because it assesses only the exposed split surface. These method-specific limitations should guide both clinical interpretation and future research design.

## 1. Introduction

Non-surgical root canal retreatment is generally considered the first-line approach for managing post-treatment endodontic disease. However, retreatment outcomes may be compromised when residual microorganisms and remnants of root canal filling material persist in anatomically inaccessible regions of the root canal system, limiting disinfection and contributing to persistent apical pathology [1,2]. Consequently, effective removal of root canal filling material remains a key objective during retreatment procedures. However, complete removal is difficult to achieve in vitro and in clinical practice, particularly in complex canal anatomies [3].

Calcium silicate-based sealers are increasingly used in contemporary endodontics due to their favorable physicochemical properties and reported bioactivity. However, microscopic, physicochemical, clinical, and retreatability studies have shown that the behavior of these materials during retreatment remains clinically important, and residual remnants may persist despite the use of modern file systems and adjunctive procedures [4,5,6,7,8,9,10,11,12,13,14,15,16,17].

A reliable assessment of residual filling material is essential when comparing retreatment protocols, as different evaluation methods capture distinct physical dimensions and are influenced by method-specific limitations. Systematic reviews and laboratory studies have highlighted the importance of cone-beam computed tomography (CBCT), microcomputed tomography (micro-CT), and other imaging approaches in root canal evaluation. Moreover, surface-based microscopic methods have been used to assess exposed canal wall residues and dentinal cleanliness [18,19,20,21,22,23,24,25,26,27,28,29,30,31,32,33,34].

Digital microscopy (DGM) provides high-resolution visualization of the exposed split root surface. Nevertheless, this approach is inherently a two-dimensional, destructive method and cannot detect remnants beneath that surface. In contrast, CBCT is clinically available but susceptible to voxel-based distortions and beam-hardening artifacts. In contrast, micro-CT provides non-destructive three-dimensional measurements and is widely used as a laboratory reference method [20,21,22,23,24,25,27,28,29,30,31,32,33,34,35,36,37,38,39,40].

Clinically, overestimation of residual material may lead to unnecessary additional retreatment procedures, whereas underestimation may result in persistent infected remnants being overlooked, potentially compromising treatment outcomes. Therefore, the aim of this in vitro study was to compare the diagnostic performance of CBCT and DGM for detecting residual root canal filling material after retreatment using micro-CT as the reference standard, with analyses performed separately for the coronal, middle, and apical thirds. The null hypothesis was that there would be no difference among micro-CT, CBCT, and DGM in their ability to detect residual root canal filling material.

## 2. Materials and Methods

### 2.1. Study Design and Ethical Approval

This experimental in vitro study received ethical approval from the Local Research Ethics Committee of Damascus University (approval no. UDDS-412-22042024/SRC-3180). The manuscript was prepared according to the STARD 2015 guidelines for reporting diagnostic accuracy studies and was conducted in accordance with the Declaration of Helsinki [41]. This study was funded by Damascus University (funder No. 501100020595).

### 2.2. Sample Size Calculation

Based on data from a previous CBCT versus micro-CT study, the sample size was calculated using G*Power 3.1.9.4 (Heinrich Heine University, Düsseldorf, Germany). For a repeated-measures design, a total sample size of 15 teeth was required to detect a large effect size (f = 0.75), which was derived from differences in residual volume measurements, with 80% statistical power at a significance level of α = 0.05 [23].

### 2.3. Sample Selection and Standardization

Fifteen extracted human mandibular premolars were included. The inclusion criteria were intact teeth with mature apices, a single root, and a single canal. Teeth were extracted for orthodontic or periodontal reasons, and patients provided informed consent for research use. This tooth type was selected as a standardized model and is consistent with previously reported anatomical characterization of mandibular premolars in the same broader population [24].

Exclusion criteria included teeth with resorption, open apices, root curvature greater than 15°, or cracks. Root curvature was assessed on standardized periapical radiographs. Cracks were screened under ×2.5 magnification using a Carson handheld loupe (Carson, Ronkonkoma, NY, USA).

The specimens were decoronated with diamond disks under water cooling to obtain a standardized root length of 17 mm. The working length was established at 16 mm by inserting a #10 K-file (Shenzhen Perfect Medical Instruments Co., Ltd., Shenzhen, China) until the tip was visible at the apical foramen, after which the length was 1 mm. All endodontic procedures were performed by the same operator (M.A.), who had 4 years of experience in endodontic practice, following a standardized protocol to minimize procedural variability and improve consistency across specimens.

### 2.4. Canal Preparation, Obturation, and Retreatment

Access cavities were prepared using diamond burs. Canals were instrumented with MG3 GOLD rotary instruments (Shenzhen Perfect Medical Instruments Co. Ltd., Shenzhen, China) to a final size of 25/04 at 350 rpm and 3 N·cm torque.

Irrigation was performed with 2 mL of 5.25% sodium hypochlorite (Septodont, Saint-Maur-des-Fossés, France) between instruments using a 30-gauge side-vented needle (EndoA-Eze, Ultradent Products, South Jordan, UT, USA). The final irrigation mixture consisted of 20 mL of 5.25% sodium hypochlorite followed by 5 mL of 17% EDTA (Septodont, Saint-Maur-des-Fossés, France). Irrigant activation was performed using Irriflex needles (Produits Dentaires SA, Vevey, Switzerland). Canals were dried with paper points.

Canals were obturated using a single-cone technique with a gutta-percha cone matching the final preparation size (25/04) (Shenzhen Perfect Medical Instruments Co., Ltd., Shenzhen, China) and a calcium silicate-based sealer (AH Plus Bioceramic, Dentsply Sirona, Ballaigues, Switzerland) according to the manufacturer’s instructions. The obturation quality was verified via CBCT using a PaX-i3D Green unit (Vatech, Hwaseong-si, Republic of Korea). Access cavities were sealed with temporary filling material (Cavit, 3 M, Seefeld, Germany), and specimens were stored in phosphate-buffered saline for 28 days to ensure complete setting of the obturation materials [42].

Retreatment was performed with ProTaper Universal Retreatment files (Dentsply Maillefer, Ballaigues, Switzerland) at 350 rpm and 1.5 N·cm torque. D1 was used for the coronal third, D2 was used for the middle third, and D3 was advanced to the working length. Between instruments, 5 mL of 5.25% sodium hypochlorite was used to flush debris. The final irrigation mixture consisted of 10 mL of 5.25% sodium hypochlorite, 5 mL of saline, 3 mL of 17% EDTA, and 5 mL of saline. Retreatment was considered complete when the irrigant appeared clear [42].

### 2.5. Outcome Definition and Regional Standardization

The target outcome was defined as residual root canal filling material, that is, any remnant of the gutta-percha-sealer complex remaining within the canal space after retreatment. Radiopaque remnants identified on computed tomographic imaging could not be reliably separated into gutta-percha and sealer components based solely on grayscale information.

For regional analysis, the 16 mm working length was divided into three equal thirds (coronal, middle, and apical). All the measurements and scores were recorded separately for each third.

### 2.6. CBCT and Micro-CT Acquisition

After retreatment, all specimens were scanned using a CBCT device (PaX-i3D Green, Vatech, Hwaseong-si, Republic of Korea) at 90 kV, 10.2 mA, a 5 cm × 5 cm field of view, a voxel size of 0.10 mm, and an exposure time of 12.57 s. Digital Imaging and Communications in Medicine datasets were exported for analysis. Following CBCT, specimens were scanned using an IRIS PET/CT micro-CT system (Inviscan, Strasbourg, France) at 80 kVp, 2000 projections, and an isotropic voxel size of 60 µm. The same custom-made holder was used for both acquisitions to standardize the specimen orientation.

### 2.7. Image Processing and Volumetric Computation

**Figure 1:**
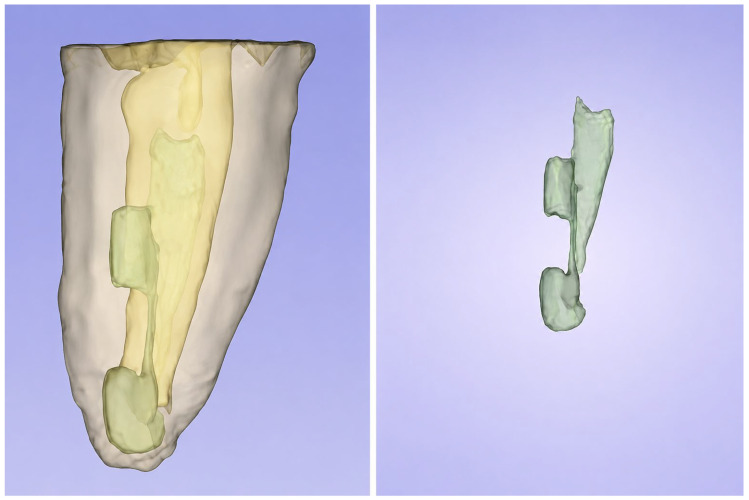
Representative segmentation of residual obturation material on CBCT using 3D Slicer. (**Left**) aligned tooth model with segmented canal space and residual material. (**Right**) isolated three-dimensional rendering of the segmented residual material.

**Figure 2:**
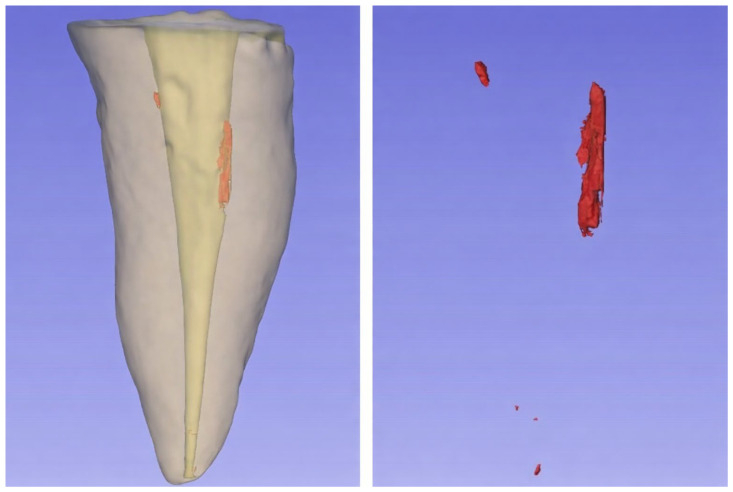
Representative segmentation of residual obturation material on micro-CT using 3D Slicer. (**Left**) aligned tooth model with segmented canal space and residual material. (**Right**) isolated three-dimensional rendering of the segmented residual material.

The CBCT and micro-CT datasets were analyzed using 3D Slicer version 5.7.0 (Brigham and Women’s Hospital, Boston, MA, USA). Representative segmentation outputs are shown in Figure 1 and Figure 2. The datasets were aligned along the long axis, cropped to the root region of interest, and segmented using grayscale thresholding with manual refinement. The postretreatment canal lumen was segmented as the radiolucent canal space, whereas residual filling material was segmented as radiopaque structures within the canal. Segments were verified in axial, coronal, and sagittal planes.

Because grayscale values differed slightly between CBCT and micro-CT datasets, threshold selection was adjusted individually for each scan based on visual identification of dentin, canal lumen, and radiopaque residual material across multiplanar views. A standardized segmentation workflow was applied by the same calibrated evaluators to improve reproducibility and consistency.

Volumes were calculated using the Segment Statistics module. For each root third, the residual volume percentage was calculated as the residual filling volume divided by the canal lumen volume multiplied by 100. Volumetric percentage outcomes were available only for CBCT and micro-CT and were compared only between these two modalities.

### 2.8. DGM and Surface Area Quantification

After micro-CT imaging, the canals were dried with paper points, and the canal orifice was protected with cotton to prevent debris entry. Two longitudinal grooves were prepared on the buccal and lingual surfaces to a depth of 0.6 mm without perforating the canal wall using a fine diamond disk under continuous water cooling. Roots were split longitudinally along the long axis with a chisel and a small mallet directed through the prepared grooves. No scraping or cleaning of the canal walls was performed after splitting.

**Figure 3:**
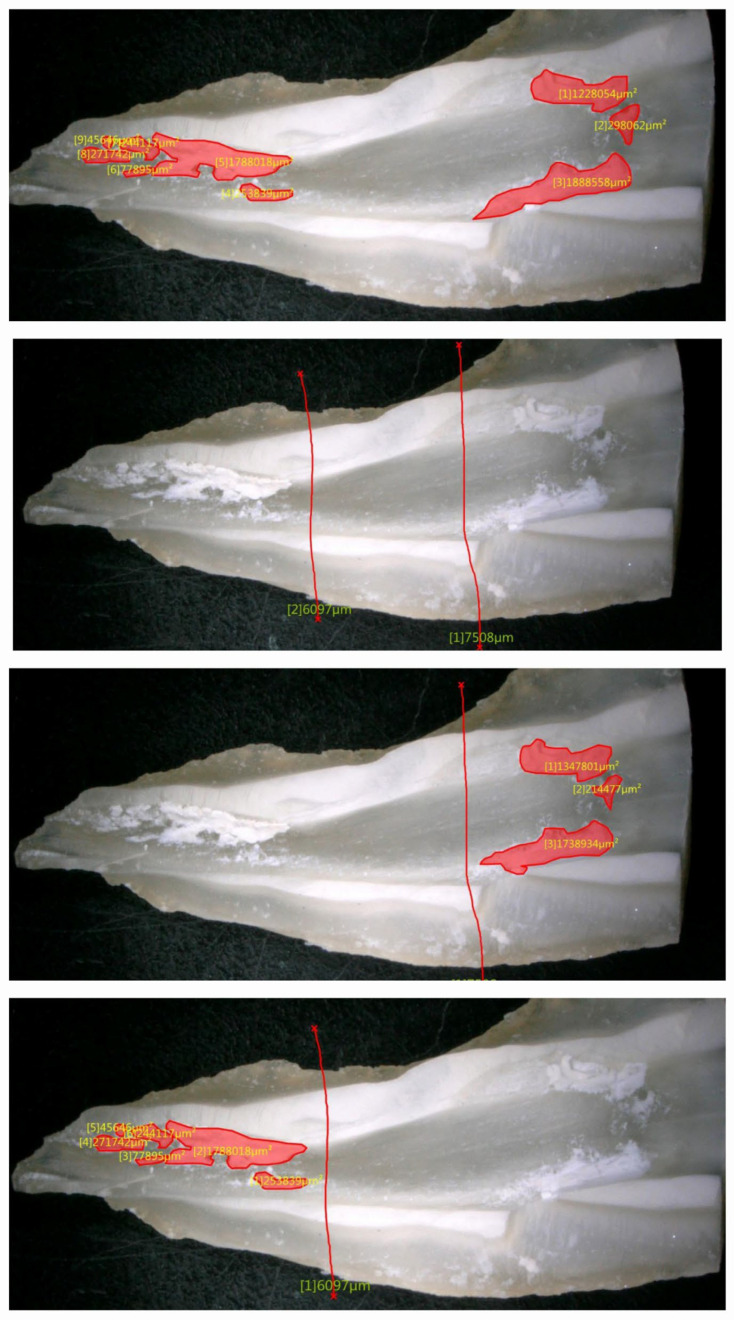
A representative DGM workflow after longitudinal splitting of the root is shown. Residual remnants were outlined on the exposed canal surface and third (in red) and measured within standardized root thirds to calculate the residual surface percentage.

The half with the best canal wall visualization (blinded to the micro-CT findings) was analyzed, while the other half was retained for documentation. Specimens were imaged using a digital microscope (VHX-5000, Keyence, Osaka, Japan). Residual filling remnants visible on the exposed canal surface were measured for each root third using the manufacturer’s software. A representative DGM workflow is shown in Figure 3. The residual surface percentage was calculated as the residual area divided by the canal surface area multiplied by 100.

DGM provides two-dimensional surface area measurements, which are not treated interchangeably with the volumetric percentages generated by CBCT or micro-CT.

### 2.9. Examiner Calibration and Measurement Reliability, Scoring, and Statistical Analysis

DGM assessments were performed by two trained evaluators (Y.A.T. and M.T.A.), and 10% of the specimens were independently reviewed by a third evaluator (S.A.). Interexaminer agreement was assessed using Cohen’s κ and was excellent (κ = 0.953, *p* ≤ 0.001).

All CBCT, micro-CT, and DGM assessments were performed independently, and the evaluators were blinded to the findings of the other imaging modalities during analysis. For the three-dimensional datasets (CBCT and micro-CT), each volumetric measurement was repeated three times, and the mean value of the three measurements was used for the final analysis. The same two calibrated evaluators who performed the DGM assessments also conducted the CBCT and micro-CT analyses to ensure consistency in image interpretation and scoring procedures.

To support diagnostic and agreement analyses without converting area and/or volume percentages into a common scale, a semiquantitative ordinal scoring system was applied to each root third. The scores were defined as follows: 0, no residual material visible; 1, trace of material, minimal remnants covering <7% of the canal wall; 2, mild, residual material covering 7–12% of the canal wall; 3, moderate, coverage of 13–18%; and 4, extensive, coverage exceeding 19% of the canal wall.

For binary detection, residual material was considered present when the score was ≥1 and absent when the score was 0. Given that this scoring system was adapted for this study, it should be considered semiquantitative and not previously validated.

The statistical analysis was performed using IBM SPSS Statistics version 26.0 (IBM Corp., Armonk, NY, USA). The data distribution was assessed using the Shapiro-Wilk test. CBCT and micro-CT residual volume percentages were compared within each third of the root using the Wilcoxon signed-rank test. The DGM surface percentages were summarized descriptively and were not pooled with volumetric measurements for direct inferential comparison. Using micro-CT data as the reference standard, the sensitivity, specificity, positive predictive value, negative predictive value, and overall accuracy were calculated for CBCT and DGM data from 2 × 2 contingency tables. Agreement was assessed using Cohen’s κ for binary detection and weighted Cohen’s κ for ordinal extent scores. The statistical significance was set at α = 0.05, and Bonferroni correction was applied only to the volumetric comparison.

## 3. Results

The inter-examiner reliability for DGM was excellent (Cohen’s κ = 0.953, *p* < 0.001).

### 3.1. Method Specific Quantitative Measurements of Residual Obturation Material

**Figure 4:**
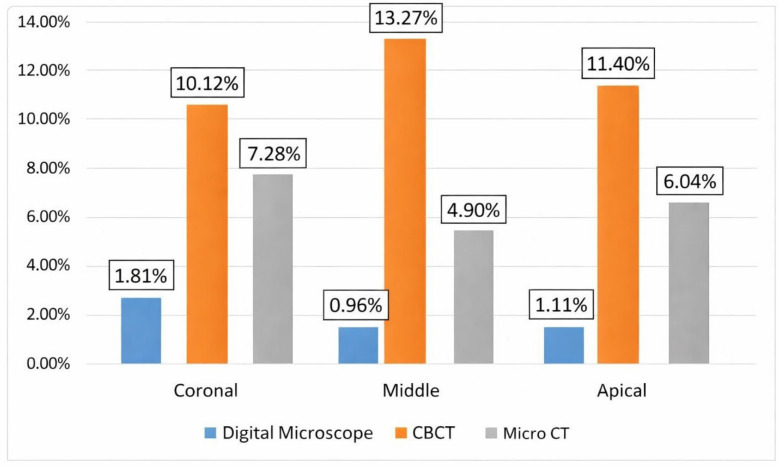
Schematic representation showing the mean percentage of residual obturation material for each evaluation method.

Table 1 and Figure 4 show the method-specific quantitative measurements of residual obturation material in the coronal, middle, and apical thirds. Digital microscopy provides a 2D surface area assessment (residual surface percentage), whereas CBCT and micro-CT provide 3D volumetric assessment (residual volume percentage). Therefore, these percentages represent different physical dimensions and are presented as method-specific outcomes, not interchangeable values.

Across thirds, digital microscopy showed low median residual surface percentages (0.34–1.52%). In contrast, CBCT showed higher median residual volume percentages (10.20–14.20%) than micro-CT (3.27–5.04%) (Table 1).

### 3.2. Paired Volumetric Comparison Between CBCT and Micro-CT

Paired volumetric comparisons (CBCT vs micro-CT) were performed for each third, with micro-CT as the reference volumetric method. CBCT showed higher median residual volume percentages than micro-CT in all thirds (Table 2). After Bonferroni correction for three comparisons (α = 0.017), the middle third difference remained statistically significant.

### 3.3. Diagnostic Accuracy of CBCT for Detecting Residual Material Using Micro CT as Reference

Diagnostic performance was evaluated for binary detection using the predefined ordinal extent scoring system, where residual present = Score ≥ 1 and residual absent = Score 0. Based on micro-CT (reference standard), residual material was present in 7/15 (46.7%) coronal thirds, 6/15 (40.0%) middle thirds, and 7/15 (46.7%) apical thirds.

CBCT demonstrated high sensitivity in the coronal third but lower specificity across thirds, indicating a tendency to classify additional thirds as positive relative to micro-CT (Table 3). Agreement (Cohen’s κ) ranged from moderate (coronal) to poor (middle and apical).

### 3.4. Diagnostic Accuracy of Digital Microscopy for Detecting Residual Material Using Micro CT as Reference

Digital microscopy demonstrated high specificity in the coronal and middle thirds, indicating few false positive findings relative to micro-CT (Table 4). However, sensitivity was low in the coronal third and absent in the middle third (no positive classifications), reflecting limited detection of residual material identified by micro-CT in these regions. In the apical third, digital microscopy demonstrated perfect detection and agreement relative to micro-CT.

## 4. Discussion

This study compared CBCT and DGM as diagnostic methods for detecting residual root canal filling material after retreatment while also analyzing volumetric and surface-based outcomes. The null hypothesis was rejected. CBCT tended to yield higher residual volume estimates than micro-CT. In contrast, DGM is highly specific but inherently limited by its two-dimensional surface-based nature.

Micro-CT remains the most appropriate laboratory reference method for this purpose because it provides nondestructive, high-resolution three-dimensional assessment of the canal space and retained material. In the present study, CBCT produced higher median residual volume percentages than micro-CT for all thirds, with a significant difference in the middle third. This pattern is consistent with studies showing that CBCT may distort the apparent extent of radiopaque remnants because of lower spatial resolution, partial-volume effects, and beam-hardening or blooming artifacts [21,22,23,24,25,27,28,29,30,31,32,33,34].

The DGM findings should not be interpreted as evidence that little residual material remained. Instead, the low surface percentages likely reflect the fact that only the exposed split surface was measured. Longitudinal splitting cannot reveal remnants embedded beneath the exposed plane, and the fracture pathway itself may determine which deposits become visible. These constraints are consistent with broader literature on surface-based microscopic assessment and with ex vivo comparisons showing that microscopic inspection may miss structures identified by volumetric imaging [17,20,26,35].

From a practical standpoint, the two evaluated methods exhibited different diagnostic trade-offs. CBCT detected more suspected remnants but produced more frequent false-positive classifications than micro-CT, especially in the middle and apical thirds. This over-estimation may lead to unnecessary retreatment attempts in clinical practice. DGM functioned more as a rule-in test: visible remnants were trustworthy, but negative findings were weak in the coronal and middle thirds. These observations align with those of comparative CBCT and micro-CT studies, ex vivo investigations of voxel-size effects, and other reports assessing the diagnostic performance of CBCT against higher-resolution reference methods [13,23,25,28,29,32,33,36,37,38,39,40].

A methodological strength of the present investigation is that DGM and tomographic measurements were intentionally analyzed as different measurement domains. Area-based surface percentages and volume-based percentages are not interchangeable, and forcing them onto a common quantitative scale can produce misleading conclusions. By separating method-specific quantitative outcomes from binary detection analysis, the physical meaning of each method was preserved while still allowing clinically interpretable comparisons.

For clinicians, the practical implication is that a positive CBCT finding (i.e., a radiopaque remnant) should be interpreted cautiously, especially in the middle and apical thirds, where our study showed significant overestimation compared with micro-CT. Relying solely on CBCT to decide whether to reinstrument or use supplementary removal techniques may lead to unnecessary procedures, increased iatrogenic risk, and extended treatment time. Conversely, when digital microscopy is available (e.g., during surgical endodontics or in laboratory assessments of retreatability), a visible remnant on the exposed canal wall is a reliable indicator of incomplete removal (high specificity). However, a negative microscopic finding does not confirm cleanliness, because remnants embedded deeper than the split surface will be missed. Therefore, in clinical practice, microscopy is best used as a rule-in tool, while CBCT should be viewed as a sensitive but nonspecific screening method that should ideally be complemented by other clinical indicators (e.g., persistent symptoms, irrigation clarity, or micro-CT in research settings).

Recent advances in artificial intelligence and deep learning-based segmentation methods may further improve the reproducibility and accuracy of endodontic imaging analysis. Emerging AI-assisted CBCT segmentation approaches have demonstrated promising performance in automated root canal and pulp space identification, potentially reducing operator dependency and improving standardization of volumetric assessment. Future studies may also compare the diagnostic accuracy of artificial intelligence systems and experienced evaluators across CBCT, micro-CT, and digital microscopy for detecting residual root canal filling material [43,44,45].

This study has several limitations. It was conducted in vitro on a relatively small number of mandibular premolars with straight single canals, and the findings may not be generalizable to more complex anatomy. Only one CBCT unit, one voxel size, and one retreatment protocol were tested. In addition, retreatment completion was determined based on the appearance of clearance of the irrigant, which is a subjective criterion and may not reliably reflect the complete removal of residual materials. Threshold-based image analysis is also sensitive to acquisition quality and operator judgment, and the exposed split surface used for DGM may not reveal all residual deposits. Moreover, mandibular premolars were intentionally selected because of their relatively consistent anatomy, which facilitated the evaluation of subtle differences among the three methods for detecting residual filling material. However, this standardized model may limit the generalizability of the findings to teeth with more complex root canal anatomies. Nevertheless, the observed pattern of overestimation by CBCT and underestimation by DGM is rooted in fundamental physical principles (voxel averaging vs. surface access) and is likely to persist even in more complex anatomies, though the magnitude of disagreement may vary.

Future investigations should include multiple retreatment strategies, broader canal morphologies, and additional imaging settings or adjunctive removal protocols to determine whether diagnostic performance improves under different experimental conditions.

## 5. Clinical Relevance

- CBCT overestimates residual root canal filling material after retreatment. Clinicians should avoid making retreatment decisions based solely on CBCT-detected radiopacities, particularly in the middle and apical thirds, without correlating findings with clinical signs.
- Digital microscopy (or surgical operating microscopes) can reliably confirm the presence of residual material when visible, but cannot rule out remnants beneath the inspected surface.
- Micro-CT remains the gold standard for laboratory research; its volumetric accuracy cannot be matched by current clinical CBCT or surface microscopy.
- When comparing retreatment protocols in future studies, investigators should clearly specify whether they are measuring surface area or volume, as these are not interchangeable.

## 6. Take-Home Messages

- **CBCT** → sensitive but overestimates remnants → don’t retreat based on CBCT alone.
- **Microscopy** → specific but misses deep remnants → positive = retreat, negative = not conclusive.
- **Micro-CT** → research standard, not clinical.

## 7. Conclusions

Within the limitations of this study, CBCT tended to yield higher residual volumetric estimates than micro-CT and showed limited specificity for binary detection, consistent with artifact-related overestimation. Digital microscopy demonstrated high specificity but variable sensitivity, reflecting its surface-based nature. Overall, micro-CT remains the most appropriate reference method for laboratory-level volumetric assessment. For clinical practice: CBCT findings of residual material should be interpreted conservatively (high false-positive risk). Visible remnants on microscopy reliably indicate incomplete removal, but negative microscopy does not confirm the absence of debris. These diagnostic characteristics must be considered when planning retreatment or interpreting endodontic research.

## Figures and Tables

**Table 1:** Method-specific quantitative outcomes of residual obturation material by root third.

Root Third	Digital Microscopy Mean (%)	Digital Microscopy SD (%)	Digital Microscopy Median (%)	CBCT Mean (%)	CBCT SD (%)	CBCT Median (%)	Micro-CT Mean (%)	Micro-CT SD (%)	Micro-CT Median (%)
Coronal	1.81	1.7	1.52	10.12	5.37	10.2	7.28	5.54	5.04
Middle	0.96	1.27	0.34	13.27	9.44	13.1	4.9	5.08	3.27
Apical	1.11	1.35	0.74	11.4	7.65	14.2	6.04	5.29	3.83

Digital microscopy: residual surface percentage; CBCT and micro-CT: residual volume percentage.

**Table 2:** Paired volumetric comparison between CBCT and micro-CT. *: statistically significant as a footnote. (residual volume percentage).

Root Third	CBCT Median Volume (%)	Micro-CT Median Volume (%)	*p*-Value
Coronal	10.2	5.04	0.648
Middle	13.1	3.27	0.002 *
Apical	14.2	3.83	0.199

**Table 3:** Diagnostic performance of CBCT for binary detection of residual obturation material using micro-CT as the reference standard.

Third of the Root Canal	Scoring System	Sensitivity	Positive Predictive Value	Negative Predictive Value	Specificity	Accuracy	Agreement		Kappa Test
Coronal	Score (0)	50.0% (95%CI: 21.5–78.5%) (4/8)	100.0% (4/4) (95%CI: 51.0–100.0%)	63.6% (7/11) (95%CI: 35.4–84.8%)	100.0% (7/7) (95%CI: 59.0–100.0%)	73.3% (11/15)	66.7% (10/15)	42.2% (19/45)	−0.031 (95% CI: −0.27 to 0.21)
	Score (1)	66.7% (2/3) (95%CI: 20.8–93.9%)	33.3% (2/6) (95%CI: 9.7–70.0%)	88.9% (8/9) (95%CI: 56.5–98.0%)	66.7% (8/12) (95%CI: 39.1–86.2%)	66.7% (10/15)			
	Score (2)	100.0% (3/3) (95%CI: 43.9–100.0%)	75.0% (3/4) (95%CI: 30.1–95.4%)	100.0% (11/11) (95%CI: 74.1–100.0%)	91.7% (11/12) (95%CI: 64.6–98.5%)	93.3% (14/15)			
	Score (3)	100.0% (1/1) (95%CI: 20.7–100%)	100.0% (1/1) (95%CI: 20.7–100.0%)	100.0% (14/14) (95%CI: 78.5–100.0%)	100.0% (14/14) (95%CI: 78.5–100.0%)	100.0% (15/15)			
	Score (4)	-	-	100.0% (15/15) (95%CI: 79.6–100.0%)	100.0% (15/15) (95%CI: 79.6–100.0%)	100.0% (15/15)			
Middle	Score (0)	33.3% (3/9) (95%CI: 12.1–64.6%)	60.0% (3/5) (95%CI: 23.1–88.2%)	40.0% (4/10) (95%CI: 16.8–68.7%)	66.7% (4/6) (95%CI: 30.0–90.3%)	46.7% (7/15)	33.3% (5/15)		0.09 (95% CI: −0.20 to 0.37)
	Score (1)	25.0% (1/4) (95%CI: 4.6–69.9%)	50.0% (1/2) (95%CI: 9.5–90.5%)	76.9% (10/13) (95%CI: 49.7–91.8%)	90.9% (10/11) (95%CI: 62.3–98.4%)	73.3% (11/15)			
	Score (2)	50.0% (1/2) (95%CI: 9.5–90.5%)	25.0% (1/4) (95%CI: 4.6–69.9%)	90.9% (10/11) (95%CI: 62.3–98.4%)	76.9% (10/13) (95%CI: 49.7–91.8%)	73.3% (11/15)			
	Score (3)	-	0.0% (0/1) (95%CI: 0.0–79.3%)	100.0% (14/14) (95%CI: 78.5–100.0%)	93.3% (14/15) (95%CI: 70.2–98.8%)	93.3% (14/15)			
	Score (4)	-	0.0% (0/3) (95%CI: 0.0–56.1%)	100.0% (12/12) (95%CI: 75.8–100.0%)	80.0% (12/15) (95%CI: 54.8–93.0%)	80.0% (12/15)			
Apical	Score (0)	25.0% (2/8) (95%CI: 7.2–59.1%)	40.0% (2/5) (95%CI: 11.8–76.9%)	40.0% (4/10) (95%CI: 16.8–68.7%)	57.1% (4/7) (95%CI: 25.0–84.2%)	40.0% (6/15)	26.6% (4/15)		0.54 (95% CI: 0.22 to 0.86)
	Score (1)	0.0% (0/4) (95%CI: 0.0–60.2%)	0.0% (0/1) (95%CI: 0.0–79.3%)	71.4% (10/14) (95%CI: 45.4–88.3%)	90.9% (10/11) (95%CI: 62.3–98.4%)	66.7% (10/15)			
	Score (2)	66.7% (2/3) (95%CI: 20.8–93.9%)	28.6% (2/7) (95%CI: 8.2–64.1%)	87.5% (7/8) (95%CI: 52.9–97.8%)	58.3% (7/12) (95%CI: 32.0–80.7%)	60.0% (9/15)			
	Score (3)	-	0.0% (0/1) (95%CI: 0.0–79.3%)	100.0%% (14/14) (95%CI: 78.5–100.0%)	93.3% (14/15) (95%CI: 70.2–98.8%)	93.3% (14/15)			
	Score (4)	-	0.0% (0/1) (95%CI: 0.0–79.3%)	100.0% (14/14) (95%CI: 78.5–100.0%)	93.3% (14/15) (95%CI: 70.2–98.8%)	93.3% (14/15)			

**Table 4:** Diagnostic performance of digital microscopy for binary detection of residual obturation material using micro-CT as the reference standard.

Third of the Root Canal	Scoring System	Sensitivity	Positive Predictive Value	Negative Predictive Value	Specificity	Accuracy	Agreement		Kappa Test
Coronal	Score (0)	100.0% (8/8) (95%CI: 67.6–100.0%)	57.1% (8/14) (95%CI: 32.6–78.6%)	100.0% (1/1) (95%CI: 20.7–100.0%)	14.3% (1/7) (95%CI: 2.6–51.3%)	60.0% (9/15)	53.3% (8/15)	55.5% (25/45)	NA (no variability in data)
	Score (1)	0.0% (0/3) (95%CI: 0.0–70.8%)	0.0% (0/1) (95%CI: 0.0–79.3%)	78.6% (11/14) (95%CI: 52.4–92.4%)	91.7% (11/12) (95%CI: 64.6–98.5%)	73.3% (11/15)			
	Score (2)	0.0% (0/3) (95%CI: 0.0–70.8%)	-	80.0% (12/15) (95%CI: 54.8–93.0%)	100.0% (12/12) (95%CI: 75.8–100.0%)	80.0% (12/15)			
	Score (3)	0.0% (0/1) (95%CI: 0.0–79.3%)	-	93.3% (14/15) (95%CI: 70.2–98.8%)	100.0% (14/14) (95%CI: 78.5–100.0%)	93.3% (14/15)			
	Score (4)	-	-	100.0% (15/15) (95%CI: 79.6–100.0%)	100.0% (15/15) (95%CI: 79.6–100.0%)	100.0% (15/15)			
Middle	Score (0)	100.0% (9/9) (95%CI: 70.1–100.0%)	60.0% (9/15) (95%CI: 35.8–80.2%)	-	0.0% (0/6) (95%CI: 0.0–39.3%)	60.0% (9/15)	60.0% (9/15)		NA (no variability in data)
	Score (1)	0.0% (0/4) (95%CI: 0.0–60.2%)	-	73.3% (11/15) (95%CI: 48.1–89.1%)	100.0% (11/11) (95%CI: 74.1–100.0%)	73.3% (11/15)			
	Score (2)	0.0% (0/2) (95%CI: 0.0–65.8%)	-	86.7% (13/15) (95%CI: 62.1–96.3%)	100.0% (13/13) (95%CI: 77.2–100.0%)	86.7% (13/15)			
	Score (3)	-	-	100.0% (15/15) (95%CI: 79.6–100.0%)	100.0% (15/15) (95%CI: 79.6–100.0%)	100.0% (15/15)			
	Score (4)	-	-	100.0% (15/15) (95%CI: 79.6–100.0%)	100.0% (15/15) (95%CI: 79.6–100.0%)	100.0% (15/15)			
Apical	Score (0)	53.3% (8/8) (95%CI: 30.1–75.2%)	100.0% (8/8) (95%CI: 67.6–100.0%)	-	-	53.3% (8/15)	53.3% (8/15)		0.045 (95%CI: −0.05 to 0.14)
	Score (1)	100.0% (4/4) (95%CI: 51.0–100.0%)	100.0% (4/4) (95%CI: 51.0–100.0%)	100.0% (11/11) (95%CI: 74.1–100.0%)	100.0% (11/11) (95%CI: 74.1–100.0%)	100.0% (15/15)			
	Score (2)	100.0% (3/3) (95%CI: 43.9–100.0%)	100.0% (3/3) (95%CI: 43.9–100.0%)	100.0% (12/12) (95%CI: 75.8–100.0%)	100.0% (12/12) (95%CI: 75.8–100.0%)	100.0% (15/15)			
	Score (3)	-	-	100.0% (15/15) (95%CI: 79.6–100.0%)	100.0% (15/15) (95%CI: 79.6–100.0%)	100.0% (15/15)			
	Score (4)	-	-	100.0% (15/15) (95%CI: 79.6–100.0%)	100.0% (15/15) (95%CI: 79.6–100.0%)	100.0% (15/15)			

## Data Availability

The raw data supporting the conclusions of this article will be made available by the authors on request.
